# Supplementary material for: Monitoring morphometric drift in lifelong learning segmentation of the spinal cord
Source: ArXiv. 2025 May 2:arXiv:2505.01364v1. Preprint. [Version 1] (PMC12306831)
Supplement: 1 [file NIHPP2505.01364V1-supplement-1.pdf]

Supplementary Material: Minimizing morphometric drift in lifelong learning segmentation of the spinal cord

This document contains the supplementary material presenting an overview of the dataset characteristics, additional plots comparing the variability in spinal cord cross-sectional area (CSA) per individual contrasts, CSA variability split across vendors, and quantitative comparison in terms of the Dice scores, relative volume error and average surface distance.

S1.1. Dataset characteristics

Table S1 contains a detailed overview of the range of image resolutions and orientations for each contrast in the dataset. Note that the images span a wide range of resolutions, especially with thick slices in the axial orientation for a few contrasts.

Table S1. *Dataset characteristics grouped by image orientation (axial, sagittal) and resolution (isotropic, anisotropic) for each contrast. Mean in-plane resolution and mean slice thickness are shown, followed by their respective minimum and maximum range of resolutions (in square brackets).*

**Contrasts**	**Isotropic**		**Anisotropic Axial Orientation**		**Anisotropic Sagittal Orientation**	
	in-plane resolution (mm^2)	slice thickness (mm)	in-plane resolution (mm^2)	slice thickness (mm)	in-plane resolution (mm^2)	slice thickness (mm)
**T1-w**	1.0 x 1.0 [1.0, 1.0]	1.0 [1.0, 1.0]	0.35 x 0.35 [0.35 x 0.35, 0.35 x 0.35]	2.54 [2.5, 5.0]	1.0 x 1.0 [1.0, 1.0]	1.0 [1.0, 1.0]
**T2-w**	0.8 x 0.8 [0.8, 0.8]	0.8 [0.8, 0.8]	0.5 x 0.5 [0.3 x 0.3, 0.8 x 1.0]	3.8 [1.0, 7.0]	0.48 x 0.48 [0.28 x 0.28, 0.96 x 0.96]	2.13 [0.8, 4.83]
**T2*-w**	–	–	0.44 x 0.44 [0.29 x 0.29,	4.93 [2.5, 9.2]	–	–

			0.5 x 0.5]			
**MT-on**	–	–	0.89 x 0.89 [0.62 x 0.62, 0.9 x 0.9]	5.06 [5.0, 9.3]	–	–
**GRE-T1w**	–	–	0.89 x 0.89 [0.68 x 0.68, 0.9 x 0.9]	5.0 [5.0, 5.0]	–	–
**DWI**	–	–	0.89 x 0.89 [0.34 x 0.34, 1.0 x 1.0]	5.0 [4.91, 5.0]	–	–
**PSIR**	–	–	–	–	0.69 x 0.69 [0.67 x 0.67, 0.69 x 0.69]	3.0 [3.0, 3.0]
**STIR**	–	–	–	–	0.7 x 0.7 [0.7 x 0.7, 0.7 x 0.7]	3.0 [3.0, 3.0]
**MP2RAGE UNIT1**	1.0 x 1.0 [1.0, 1.0]	1.0 [1.0, 1.0]	–	–		

S1.2. CSA variability across individual contrasts

Figure S1 shows the variability in the spinal cord CSA across six contrasts on the test set (*n=49*; n_{vol}*=294*) of the spine-generic multi-subject database (Cohen-Adad et al., 2021a) between three methods: (i) `sct_deepseg_sc` (Gros et al., 2019), (ii) our previous model, `contrast-agnostic_v2.0` (Bédard et al., 2025), and the proposed model, `contrast-agnostic_v3.0`. Compared to the previous version (`v2.0`), our proposed model (`v3.0`) achieves a similar CSA variability on the test set of healthy participants despite being trained on heterogeneous data containing new contrasts and several pathologies.

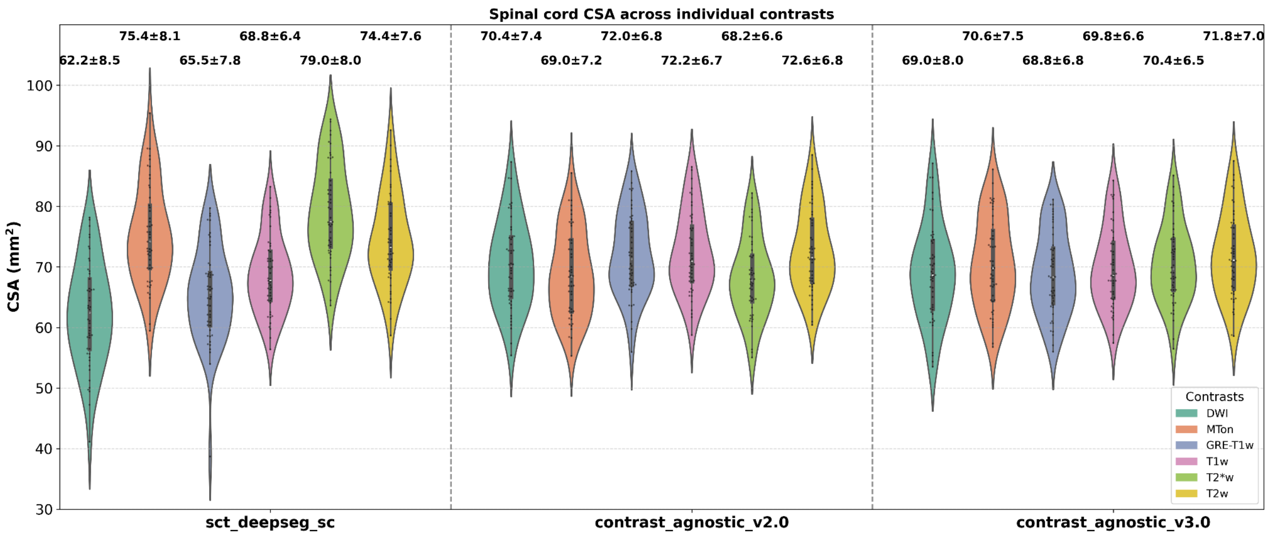

Figure S1. *Variability in spinal cord CSA across 6 contrasts compared with existing automatic segmentation methods on a test set of healthy participants (n=49). Even after the addition of new pathologies and contrasts to the training set, CSA variability achieved by the proposed* `contrast-agnostic_v3.0` *model remains similar to our previous model* `contrast-agnostic_v2.0` *(trained only on a healthy participants database) and shows a substantial improvement over* `sct_deepseg_sc`.

S1.3. CSA variability across scanner manufacturers

In this section, we evaluate the variability in the CSA measurements for a *single subject* across different scanner manufacturers. We used the spine-generic `data-single-subject` dataset (Cohen-Adad et al., 2021), which includes cervical spinal cord scans in a single healthy participant using six contrasts (T2w, T1w, T2*w, MT-on, GRE-T1w, and DWI) across 15 sites with 3 scanner vendors (GE; *n=4*, Philips; *n=4*, Siemens; *n=7*). As with the previous evaluations, we compared three methods: `sct_deepseg_sc` (Gros et al., 2019), `contrast_agnostic_v2.0` (Bédard et al., 2025), and the proposed `contrast_agnostic_v3.0`, for contrasts and sites. In all comparisons, the spinal cord segmentations were obtained independently for each of the above methods, and the vertebral levels were identified using `sct_label_vertebrae`. Then, we calculated the CSA averaged across C2-C3 vertebral levels and computed its standard deviation (STD) across scanner manufacturers.

It is important to stress that all data points represent the *same* participant. each of the 6 contrasts comparing the two segmentation methods across all 15 sites. **Figure S2** presents the CSA STD across 6 contrasts per site for both segmentation methods, separated per MRI vendor. The STD using the `contrast_agnostic_v3.0` method yields a lower STD than when using `sct_deepseg_sc` for segmentation, and is very similar to `contrast_agnostic_v2.0`.

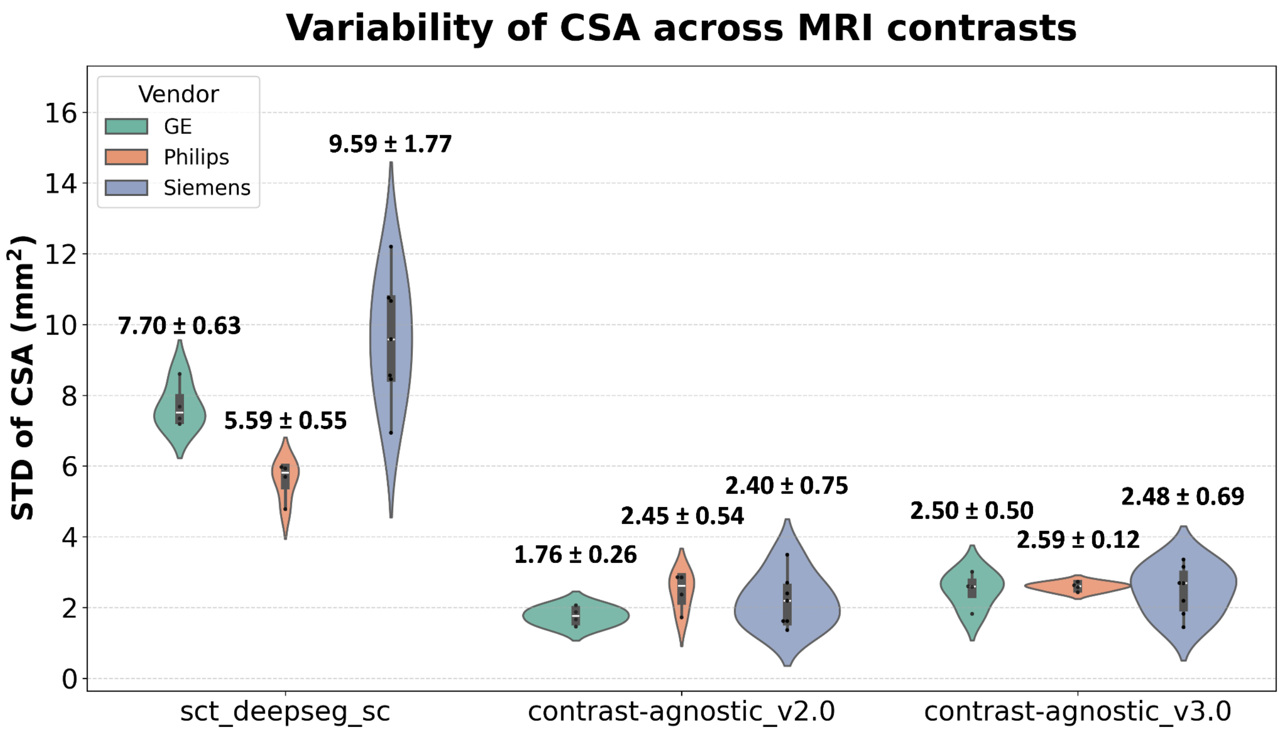

Figure S2. *Variability of spinal cord CSA across contrasts separated per vendor for segmentations generated with sct_deepseg_sc (Gros et al., 2019), contrast-agnostic_v2.0 (Bédard et al., 2025) and contrast-agnostic_v3.0 (proposed) segmentation and contrast-agnostic of the same participant scanned across 15 different MRI sites. Each dot represents one site; mean and standard deviation are presented above.*

S1.4. CSA variability with recursively generated labels

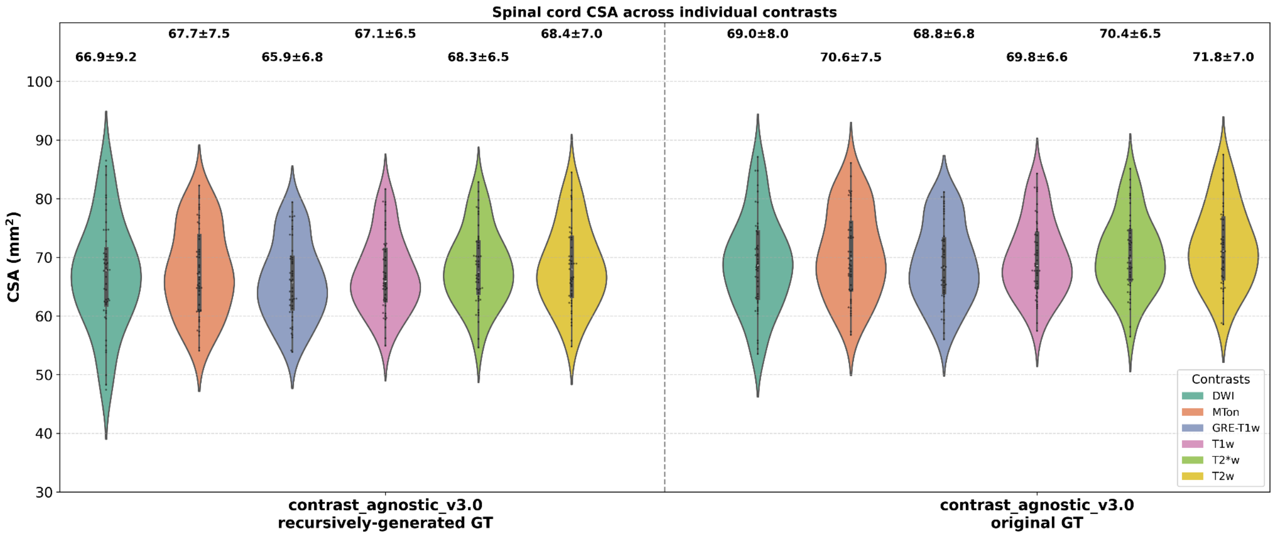

Figure S3. *Variability in spinal cord CSA across 6 contrasts on a test set of healthy participants (n=49) compared between the models trained with the: (i) original distribution of GT masks created*

from a mix of manual annotations and automatic segmentation methods, and (ii) GT masks regenerated with contrast_agnostic_v3.0 model without any manual corrections. The model trained on recursively generated GT masks achieved lower average CSA per contrast compared to the model trained on the original distribution of GT masks on all contrasts.

Figure S3 plots the average CSA per contrast for the ablation study, comparing the downstream effect of training the contrast_agnostic_v3.0 model on the original distribution of GT masks and the masks generated recursively without any manual correction.
